# Changing Bacterial Profiles and Antibiotic Susceptibility Patterns in Spontaneous Ascites Infection: A Shift in Empiric Combination Antibiotic Therapy?

**DOI:** 10.3390/antibiotics14111132

**Published:** 2025-11-08

**Authors:** Ceren Kaplankıran, Ender Serin

**Affiliations:** 1 Department of Immunology and Allergy Diseases, Ankara Bilkent City Hospital, Ankara 06800, Turkey; 2 Department of Gastroenterology, Adana Medical and Research Center, Baskent University Faculty of Medicine, Adana 01250, Turkey; eserin67@yahoo.com

**Keywords:** spontaneous ascites infection, SBP, cirrhosis, antibiotic resistance, bacterial diversity

## Abstract

**Background/Objectives:** This study aimed to determine bacteria that grow in spontaneous ascites infection and their antibiotic susceptibility. **Materials and Methods:** A total of 291 cases of cirrhosis-related ascites were retrospectively analyzed between January 2007 and September 2015. Simple ascites, spontaneous ascites infection, spontaneous bacterial peritonitis, and culture results were recorded. Due to increasing antibiotic resistance, theoretically appropriate antibiotics were paired, and their susceptibility was evaluated in order to review our empirical antibiotic choices and evaluate new empirical treatment options. **Results:** In all cultures, 48.1% were Gram-positive cocci and 50.6% were Gram-negative bacilli. Multidrug-resistant bacteria grew in 16 cultures (22.2%). The most common bacterium was *Escherichia coli*, and the second most common bacteria were coagulase-negative staphylococci (CoNS). According to all culture data, the rate of antibiotic susceptibility was third- and fourth-generation cephalosporins between 76.4 and 68%, 68% for ampicillin-sulbactam, 81.8% for piperacillin-tazobactam, 66% for ciprofloxacin, 71% for levofloxacin, 30% for tetracycline, and 45% for penicillin. Beta-lactam group antibiotic susceptibility of CoNS was 25% for penicillin and 53% for ampicillin-sulbactam; 52% had methicillin resistance. The rate of resistance to quinolones was 35%. Multidrug resistance was detected in 40% of CoNS (eight cases). When theoretically appropriate antibiotics were paired, combination therapy increased the susceptibility rates. **Conclusions:** The increase in Gram-positive infections and resistance to other antibiotic groups indicates empirical antibiotic selection based on local microbial culture results and antibiotic susceptibility. Our results are crucial to allowing for more rational and successful use of antibiotics.

## 1. Introduction

Spontaneous ascites infection (SAI) is a common and life-threatening complication of decompensated cirrhosis. It is an important marker of progression in patients with end-stage cirrhosis [1]. It accounts for 5 to 30% of all infections in patients with cirrhosis. Spontaneous ascitic infection occurs as a result of bacteremia caused by microorganisms colonizing the urinary and respiratory system, hypovolemic shock, mucosal ischemia due to vasoconstricting drugs, increased intestinal mucosal permeability, malnutrition, and invasive procedures (catheterization, endoscopy, esophageal sclerotherapy).

Early diagnosis and early treatment are important in terms of mortality in spontaneous bacterial peritonitis (SBP). Despite current efforts to reduce the mortality rate, the rates are still between 16% and 52% [2,3]. Spontaneous ascitic infection can develop in all cirrhotic patients with ascites. The most common bacteria are *Escherichia coli* (40–55%), *K. pneumoniae*, *Streptococcus pneumoniae*, and other streptococci [2]. Previous hospitalizations, nosocomial infections, and the use of prophylactic antibiotics have been reported to be associated with an increase in resistant bacterial infections in cirrhotic patients. Over the past decade, multiple centers have documented a rising prevalence of Gram-positive infections and multidrug-resistant (MDR) pathogens among cirrhotic patients [3,4]. These changes in bacterial profiles and resistance concern the efficacy of empirical treatment [5]. Recently, the examination of an increase in spontaneous ascites infection, microorganism diversity, and antibiotic susceptibility is necessary to determine treatment protocols [6]. Due to changing resistance rates, cephalosporins cannot be suggested for empirical treatment of healthcare-related and nosocomial SBP [7]. Over the last decade, increasing resistance in microbial patterns, for empirical antibiotic selection based on local microbial culture results and antibiotic susceptibility, has been crucial to allowing for more rational and successful use of antibiotics [4]. The shifting microbiome, characterized by an increasing proportion of Gram-positive isolates, indicates that empirical cephalosporin choices for SBP should be re-evaluated, as these agents may no longer provide adequate coverage.

This study aimed to determine bacteria that grow in spontaneous ascites infection and their antibiotic susceptibility, compare them with those reported in previous studies, determine empirical treatment protocols according to the bacterial diversity in our center, and determine whether conventional empirical treatment protocols are still effective.

## 2. Results

A total of 291 cases with cirrhosis-related ascites were included in the study. The cases were divided into two groups: growth or non-growth in ascitic fluid culture. Growth was detected in 72 (24.7%) of the ascites samples obtained from 291 cases and in 72 (66%) of those diagnosed with SAI. In SAI subgroups, 37 cases were diagnosed as culture-negative neutrocytic ascites (CNNA), 23 cases as monomicrobial non-neutrocytic bacterascites (MNBA), and 49 cases as spontaneous bacterial peritonitis (SBP). Table 1 shows the demographic and clinical data of the study.

Taking previous episodes into account, eleven (3.8%) cases hospitalized with recurrent spontaneous ascites infection who were culture positive were identified to have a second episode, five (1.7%) were identified to have a third episode, and two (0.7%) were identified to have a fourth episode; only seven of these cases were hospitalized due to SBP. The number of patients hospitalized with recurrent episodes due to CNNA and MNBA was higher than those hospitalized with SBP. Hospital mortality among all ascites cases was 33 (11.3%), of which 16 (5.4%) were due to SBP, 4 due to CNNA, and 2 due to MNBA. Hospital mortality (33%) was detected in only 16 of 49 cases in the SBP group.

When ascites culture results were evaluated, the 3 most common bacteria were *Escherichia coli* (*E. coli*) (*n* = 28, 34.5%), coagulase-negative staphylococcus (CoNS) (*n* = 20, 24.6%), and *Enterococci* (*n* = 8, 9.8%). When cases with SBP were evaluated separately, Child C was determined in 38 cases (77.6%) and Child B in 11 cases (22.4%), while Child A was not determined in any case. When SBP culture results were evaluated, the three most common microorganisms were *E. coli* (*n* = 23, 46.9%), CoNS (*n* = 7, 14.3%), and *Enterococcus* (*n* = 5, 10.2%). The most common microorganisms and their rates were similar in the SBP and MNBA groups. However, only in the MNBA group, the microorganisms most frequently found were coagulase-negative Staphylococcus instead of *E. coli*, with a rate of 56% in 13 positive culture cases. Nine of the cultures with growth were polymicrobial; one case was in the SBP group, and eight cases were in the MNBA. The rates of bacteria grown in SBP are shown in Table 2.

Spontaneous bacterial peritonitis, culture-negative neutrocytic ascites, and monomicrobial non-neutrocytic bacterascites groups were gathered in a single group and compared with patients with simple ascites. When demographic characteristics of the groups were analyzed, no significant difference was found in terms of age and Child–Pugh score. HBV and Budd–Chiari rates were higher in the simple ascites group compared to the SAI group.

In all cultures, 48.1% (37) were Gram-positive cocci and 50.6% (38) were Gram-negative bacilli. Multidrug-resistant bacteria grew in 16 cultures (22%). Vancomycin-resistant Enterococci and carbapenemase-producing bacteria were not detected in any culture. Most of the MDR bacteria (68.7%, 11 cultures in total) grew in the SBP group. Hospital mortality was seen in seven (43%) MDR bacteria, and all of them were spontaneous bacterial peritonitis. The remaining nine cases (68.7%) responded to parenteral antibiotic treatment. Table 3 shows the rates of MDR bacteria.

All Gram-positive bacteria were 100% susceptible to teicoplanin and vancomycin; moreover, the most susceptible antibiotics were imipenem (96.5%), meropenem (93.5%), cefoperazone-sulbactam (96.2%), and amikacin (90.3%), with a susceptibility of 90% and above, respectively. Almost all antibiotics had a susceptibility above 60%. In our clinic, third- and fourth-generation cephalosporins are the most commonly used empirical treatment agents. According to all culture data, the rates of antibiotic susceptibility were as follows: third- and fourth-generation cephalosporins between 76.4 and 68% (ceftriaxone 70%, ceftazidime 66%, cefuroxime 74%, cefazolin 68%, cefotaxime 68.9%, and cefepime 76.4%), ampicillin sulbactam 68%, piperacillin-tazobactam 81.8%, ciprofloxacin 66%, levofloxacin 71%, tetracycline 30%, and penicillin 45%.

The most frequently grown bacteria, *E. coli*, was 100% susceptible to imipenem and meropenem, third- and fourth-generation cephalosporins between 70.8 and 65% (cefazolin 65%, cefotaxime 68%, cefepime 68%, cefuroxime 69.5%, ceftriaxone 69%, ceftazidime 70.8%), 96.2% to cefoperazone-sulbactam, 85.7% to piperacillin-tazobactam, 71% to ciprofloxacin, and 75% to levofloxacin. Multidrug resistance was detected in five cases (17%) of *E. coli*. The susceptibility of CoNS, which was the second most frequently grown organism and the most susceptible to antibiotics, was between 100 and 70%. Among the beta-lactam group antibiotics, penicillin had a susceptibility of 25%; ampicillin-sulbactam had a susceptibility of 53%; methicillin resistance was detected at a rate of 52%. Susceptibility to quinolones was 65%. Multidrug resistance was detected in eight cases (40%) of CoNS.

**Figure 1:**
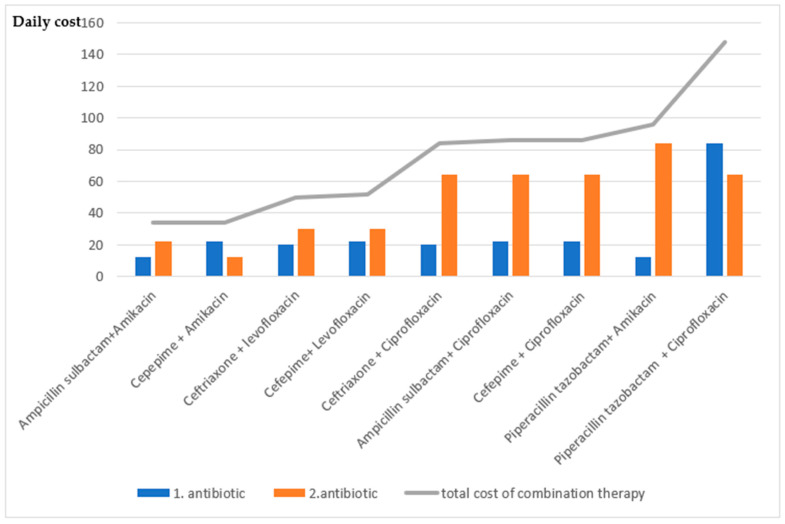
Calculation of the cost of theoretical antibiotic combinations (The cost was determined based on the average daily cost in Turkish lira for the total daily parenteral dose. Based on 2016 pricing data from the Turkish Medicines and Medical Devices Agency (TITCK). https://titck.gov.tr/dinamikmodul/100, accessed on 15 January 2016).

Due to increasing antibiotic resistance, theoretically appropriate antibiotics were paired, and their susceptibility was evaluated in order to review our empirical antibiotic choices and evaluate new empirical treatment options. The susceptibility rates were calculated theoretically. Combined susceptibility was defined as the proportion of isolates that were susceptible to at least one of the antibiotics within the tested pair. It was observed that starting with combination therapy increased the susceptibility rates. The susceptibility of glycopeptide group antibiotics in Gram-positive infections was 100% and no resistance was shown in any case in our study. However, since they had no effect on Gram-negative bacteria, combination therapy did not increase susceptibility in patients with Gram-negative culture growth. Table 4 shows the single and combination treatment of commonly used antibiotics. The costs of one-day parenteral use of antibiotics are shown in Figure 1.

In our study, the effect of cirrhosis etiology on mortality in SAI was insignificant. We found that mortality increased as the Child–Pugh score increased in SAI (*p* < 0.0001). It was found that SAI was associated with shorter survival, but SBP had no statistically significant effect. Among all cases with ascites, 11 (3.8%) experienced a second episode, 5 (1.7%) a third, and 2 (0.7%) a fourth episode. It was found that having more than one episode significantly reduced survival time (*p* < 0.0001). Child–Pugh score (*p* = 0.037) and advanced age were found to reduce survival. The median survival time of the patients who participated in the study was 4 months. The mean survival time of all cases was 22 ± 2.3 months. 291 cases in the study, 73 (25.1%) were still alive. In the 1–102-month follow-up of the patients, the mortality rate was 91.6% for SBP, 91.3% for MNBA, 78.3% for CNNA, 67.4% for simple ascites, and 86.2% for SAI. Factors affecting mortality are shown in Table 5.

## 3. Discussion

In this single-center study, *E. coli* was identified as the most common isolate in our ascitic culture results, followed by CoNS, supporting the recent rise in Gram-positive infections. We also confirmed resistance to third- and fourth-generation cephalosporins, which are among the most commonly used empirical agents, whereas our theoretical antibiotic combinations showed increased susceptibility rates. Our mortality rate among patients with MDR infections was high and comparable to that observed in sepsis. Our findings were consistent with recent reports highlighting the increasing burden of Gram-positive and MDR infections, supporting that cephalosporins are no longer recommended for empirical treatment in patients with MDR risk factors, as well as in healthcare-associated and nosocomial SBP cases [7,8]. Our results further emphasize the need for empirical therapy guided by local antimicrobial susceptibility data.

Spontaneous bacterial peritonitis is an important complication of cirrhosis, with a mortality rate of 16–52% [9,10]. Initial empirical treatment should cover all possible pathogens and should be administered in sufficient concentration to tissues that are thought to be the source of infection. Spontaneous ascites infection should be considered an abdominal sepsis, and the bacterial susceptibility of the antibiotic to be selected for empirical treatment in abdominal sepsis should be above 80% [11].

There is no growth in ascites culture in 60% of cases with symptoms clinically compatible with SBP, and those with high ascites PMNL count [12]. Of the 291 cases included in our study, 109 (37.4%) had SAI and 72 (24.7%) had growth. Growth was detected in only 72 (66%) of those diagnosed with SAI. Due to the absence of typical clinical features of SAI and sometimes the absence of clinical symptoms, diagnostic paracentesis is recommended for cirrhotic patients hospitalized for any reason [13]. Based on this indication, the simple ascites group was found to be larger in our study because ascites cell counts and cultures were sent from the patients, although there is no infection clinic. In our study, SBP constituted the largest group among SAI, followed by CNNA.

One of the reasons for the low rate of culture growth compared to the literature findings may be that the simple ascites group in our study consisted of a higher number of cases, the patients may have been receiving prophylactic antibiotics for SBP, or the patients may have eliminated the causative microorganism at the time of presentation, despite the high number of PMNL count [14]. In our study, Gram-positive cocci were detected at a rate of 48.1% (37 cases) and Gram-negative bacilli at a rate of 50.6% (38 cases). Similar to previous studies, *E. coli* was the most frequently grown microorganism with a rate of 34.5%; the second most frequently grown bacteria were CoNS with a rate of 24.6% [15,16]. As in our study, it has been determined that the ascitic culture growth of *Staphylococcus epidermidis* has increased in recent studies [17,18]. The increase in CoNS growth in our study may be due to interventional procedures (paracentesis, endoscopic interventions, and central venous catheterization). In previous studies, an increase in Gram-positive bacterial infections has been reported due to interventional procedures during hospitalization, supporting our idea [17].

In intestinal flora studies, differences have been reported in cirrhotic patients compared to healthy people [18]. Considering the role of bacterial translocation and bacterial over-reproduction in the development of SBP, the change in intestinal flora will directly affect the bacterial diversity in culture. It can also be predicted that the change in intestinal flora resulting from the frequent use of oral laxatives and prophylactic antibiotics (rifaximin, quinolones, etc.) in cirrhotic patients will lead to an increase in Gram-positive bacteria in culture. In our study, it was not known whether the patients had received antibiotic prophylaxis, used oral laxatives, had been hospitalized recently, had a history of interventional procedures, or which antibiotics were used in previous ascites infection. Likewise, recently, it has been observed that antibiotic resistance in Gram-positive infections against quinolones, which are frequently used in primary and secondary SBP prophylaxis, has also increased [19,20]. The increase in Gram-positive cocci growth reported in previous studies is similar to the finding in our clinic. This may be considered evidence that the diversity and resistance of microorganisms in SAI should be reviewed over the years.

Polymicrobial growth had no effect on survival time. The reason for insignificance was considered to be the fact that there were 9 cases in total. Of the nine cases, eight were diagnosed with MNBA (representing a polymicrobial MNBA rate of 34.8%) and one with SBP. Although MNBA is typically monomicrobial, certain polymicrobial cases in our cohort were included after excluding secondary peritonitis and contamination, as they exhibited clinical signs of infection with PMNL counts below 250 cells/mm^3^ and negative follow-up cultures. The proportion of MNBA cases was higher than in most previous cohorts; however, Oey et al. similarly reported a 19% polymicrobial rate, suggesting that such findings may reflect early infection or bacterial colonization rather than contamination or secondary peritonitis [21].

In our study, quinolone susceptibility of *E. coli* and CoNS was 65–70%, lower than the other antibiotics. In a study conducted by Yakar et al. with cirrhotic patients with ascites admitted to our clinic between January 2000 and August 2006, quinolone susceptibility was similar in *E. coli* but higher in CoNS, with a rate of 90% [22]. This supports that resistance to quinolones, which are recommended for secondary prophylaxis and frequently used in the empirical treatment of SAI, has increased over time [23,24]. This developing antibiotic resistance has raised the necessity of discovering new prophylactic antibiotics.

The current approach in the treatment and primary and secondary prophylaxis of SBP is still third- and fourth-generation cephalosporins and quinolones [12,13]. In our clinic, third- and fourth-generation cephalosporins were the first choice of treatment for SBP. Nowadays, the increasing number of broad-spectrum beta-lactamase-producing *E. coli*, methicillin-resistant *Staphylococcus aureus*, quinolone resistance that develops following norfloxacin prophylaxis, and the increase in resistant enterococcal infections are the reasons for the failure of empirical treatment [23,25]. It has been shown that those with third-generation cephalosporin-resistant bacterial growth have a higher mortality rate than antibiogram-susceptible patients [26,27]. In studies, it has been shown that bacterial diversity differs in infections in cirrhotic patients and that MDR infections have increased [3,8,28]. Broad-spectrum antibiotics are recommended in patients with risk factors, including nosocomial infections, prior norfloxacin prophylaxis, a history of the use of beta-lactam group antibiotics in the last 12 weeks, and a history of MDR infections [29]. Piperacillin–tazobactam alone or a combination of glycopeptide and carbapenem antibiotics is recommended for empirical treatment of patients with risk factors for MDR infections [8,30]. In our study, more than 20% resistance has developed to most of the third- and fourth-generation cephalosporins; we found that multidrug-resistant MDR bacteria in 16 cultures (22.2%). According to the most recent European Association for the Study of the Liver (EASL, 2018) and American Association for the Study of Liver Diseases (AASLD, 2021) clinical practice guidelines, empirical antibiotic therapy for SBP should be guided by local antimicrobial resistance patterns [31,32]. Third-generation cephalosporins, such as cefotaxime or ceftriaxone, remain the recommended first-line agents for community-acquired SBP. However, their efficacy has markedly decreased in healthcare-associated and nosocomial infections due to the increasing prevalence of MDR bacteria. In such cases, broader-spectrum antibiotic regimens—including carbapenems, with or without glycopeptides or linezolid—are advised [31,32]. Consistent with these recommendations, our study demonstrated high resistance rates to third- and fourth-generation cephalosporins. These findings underscore the need for locally adapted empirical treatment strategies and align with both guidelines’ emphasis on surveillance-based antibiotic selection rather than a universal empirical approach.

In our study, the survival rate was 91.6% in SBP, 91.3% in MNBA, and 78.3% in CNNA. Close survival rates may be due to the presence of other complications of cirrhosis that may affect mortality, non-cirrhotic causes, and prolonged follow-up of some of the patients. In our study, it was not known whether these factors affected mortality. In studies, the effect of the Child–Pugh score on mortality in the development of SBP has been shown [16,33]. In line with the literature, this suggests that severe cirrhosis is a facilitating factor in the development of SAI [15,33]. In our study, it was found that SAI was associated with shorter survival, but SBP had no statistically significant effect. The fact that only SAI was found to have a significant effect on survival time was attributed to the low number of SBP cases among SAI subgroups.

When hospital mortality rates were evaluated, hospital mortality was 11.3% (33 cases) in all ascites cirrhosis cases, of which 5.4% (16 cases) were SBP, 1.3% (4 cases) CNNA, and 0.6% (2 cases) MNBA. Hospital mortality was found to be 33% (16 cases) only in the SBP group (48 cases). Hospital mortality was high in SBP. The majority of MDR bacteria were also seen in SBP, and hospital mortality was seen in 43% of those with MDR bacteria; all of them were SBP. The reason for the high hospital mortality in SBP may be the changing antibiotic resistance and decreasing efficacy of empirical treatments. Appropriate antibiotic treatment alone does not reduce mortality, but the development of renal failure has a very important effect on mortality. For this reason, broad-spectrum antibiotics are recommended in patients with certain risk factors mentioned previously. According to these risk factors, the empirical treatment for reducing hospital mortality can be customized. However, there are reservations about the initiation of these antibiotics due to the development of renal toxicity, increased costs, and the widespread use of broad-spectrum antibiotics leading to an increase in MDR bacteria. In this study, we evaluated the susceptibility of antibiotics for which there is insufficient data on their use in the clinic, but which may be appropriate when used in combination. It was found that dual combination therapy theoretically increased susceptibility in empirical treatment despite increased costs.

When the costs of combined antibiotic therapies are evaluated, the combination with third- and fourth-generation cephalosporins is less costly compared to other combinations. Cephalosporin combinations with fluoroquinolones significantly increase the costs. Although the efficacy of piperacillin-tazobactam treatment is high, it increases cost in single and combined treatments. The susceptibility of glycopeptide group antibiotics in Gram-positive infections was determined to be 100% and no resistance was detected in any case in our study. Nevertheless, it was found that the use of a glycopeptide antibiotic combination did not increase the susceptibility of Gram-negative bacterial growth in dual combinations. Due to a shifting microbiome, Gram-positive cultures were shown to have cephalosporin resistance in SBP treatment [5]. In a multicenter study, the prevalence of bacteria increased from 29% to 38% during 2011–2018 [3]. In our study, the hospital mortality rate in MDR bacteria was 43% (7 cases), which was similar to the hospital mortality rate in sepsis (30–50%). This suggests that it may be useful to prefer empirical combination therapy in patients at risk of MDR. Although 43% of patients with MDR had hospital mortality in our study, treatment failure in these patients cannot be explained only by inappropriate empirical antibiotic selection, but other factors affecting mortality should also be taken into account.

This study has several limitations. It was conducted at a single center with a retrospective design, which may limit the external validity and applicability of the findings to other clinical settings. Factors such as prior antibiotic exposure, hospitalization history, recent invasive procedures, and prophylactic antibiotic use may have influenced both culture results and the development of resistance; however, these variables were not systematically recorded. Consequently, the potential contribution of these factors to the observed increase in Gram-positive cocci and MDR rates could not be fully evaluated. Moreover, other cirrhosis-related complications were not assessed, and the absence of long-term mortality follow-up restricted the identification of additional factors influencing survival. Despite these limitations, the study provides valuable real-world data on microbial distribution and resistance patterns in spontaneous ascitic infections.

## 4. Materials and Methods

In this single-center, retrospective study, 291 cases of ascites diagnosed with cirrhosis based on clinical, laboratory, histological, and ultrasonographic findings were analyzed. These cases applied to the Gastroenterology Department of Baskent University Adana Medical and Research Center between January 2007 and September 2015. All cases included in the study were evaluated in terms of demographic characteristics and etiologies, and their data were recorded.

Ascites samples obtained from all patients were inoculated in blood culture flasks, and all samples were portal hypertensive ascites. All patients were recorded as having simple ascites and SAI. Patients were diagnosed with SAI if the ascites polymorphonuclear leukocyte (PMNL) count was 250 cells/mm^3^ or more. Culture positivity was not required for SAI. SAI subgroups were recorded as culture-negative neutrocytic ascites (CNNA), monomicrobial non-neutrocytic bacterascites (MNBA), and spontaneous bacterial peritonitis (SBP). CNNA was defined as a negative ascites fluid culture with PMNL count of 250 cells/mm^3^ or more, no antibiotic treatment in the preceding 30 days, and the absence of an intra-abdominal source of infection that would explain the increase in ascites fluid PMNL count. Presence of a positive ascites culture with PMNL count less than 250 cells/mm^3^ was defined as MNBA. SBP was diagnosed as a positive ascites culture, ascites PMNL count of 250 cells/mm^3^ or more.

Ascitic fluid samples were inoculated into blood culture bottles and incubated in the BACTEC 9240 automated system (Becton Dickinson, Franklin Lakes, MD, USA). Subsequent subcultures were performed on MacConkey agar and chocolate agar media. Identification of Gram-positive cocci was based on catalase, coagulase, growth in 6.5% NaCl, and esculin hydrolysis tests. For Gram-negative bacteria, isolates were characterized by growth on Triple Sugar Iron (TSI) agar and tested for lysine and ornithine decarboxylase, Voges–Proskauer (VP), methyl red, phenylalanine deaminase, and indole reactions. Bacteria that could not be identified by conventional biochemical tests were analyzed using the BBL Crystal automated system (Becton Dickinson, Franklin Lakes, MD, USA). Antimicrobial susceptibility testing was performed using the Kirby–Bauer disk diffusion method (Oxoid Ltd., Basingstoke, UK) in accordance with the Clinical and Laboratory Standards Institute (CLSI) guidelines. Cases with repeated paracentesis showing no increase in ascitic PMN counts, absence of clinical signs of infection, and late BACTEC signalization (beyond 72 h) were interpreted as contamination (CoNS, *Corynebacterium* spp., *Bacillus* spp. (non-*anthracis*), *Micrococcus* spp., and *Cutibacterium acnes*) and were classified as simple ascites rather than spontaneous ascitic infection. Polymicrobial culture results were recorded as SBP and MNBA after excluding secondary bacterial peritonitis. Culture sample collections after hospitalization were taken, and the defined sample duration was <24 h, 24–48 h, 48–72 h, and >72 h. In SBP cases, available data in terms of 3rd-day control paracentesis cell count and PMNL samples were recorded. The unit of analysis was infection episodes rather than unique patients. Each episode was analyzed separately, as microbiological characteristics and antimicrobial susceptibility patterns were specific to each infection event.

Each patient’s sex, age, cirrhosis etiology, Child–Pugh score, survival time, ascites cell count, history of recurrent peritonitis episodes, hospital mortality, and nosocomial infections were recorded. Cirrhosis etiology groups defined hepatitis B, hepatitis C, alcoholic liver disease, primary biliary cirrhosis, Budd–Chiari, cryptogenic, and other reasons. Cases with metabolic comorbidities that predicted nonalcoholic liver disease were defined as cryptogenic, since not all cases had a histopathological diagnosis. Patients with secondary bacterial peritonitis and hepatocellular carcinoma accompanying cirrhosis were excluded. Information on prior antibiotic exposure, hospitalization history, other cirrhosis-related complications, and recent invasive procedures was not collected in this study and, therefore, was not included in the statistical analysis.

### Statistical Analyses

All statistical analyses were performed using IBM SPSS Statistics, version 17.0 (IBM Corp., Armonk, NY, USA). The Shapiro–Wilk test was applied to assess the normality of continuous variables. The chi-square test or Fisher’s test was used for the comparison of categorical variables. In the inter-group comparison of continuous measurements, the Student’s *t*-test was used for parameters with normal distribution according to the number of variables, and ANOVA was used for the comparison of more than two variables. The Mann–Whitney U test was used for parameters without normal distribution, and the Kruskal–Wallis test was used for the comparison of more than two variables. The Kaplan–Meier method was used for the survival curve, and the Log-rank test was used to calculate survival differences between the groups. A *p*-value < 0.05 was considered statistically significant. No sample size calculation was performed due to the retrospective nature of the study.

## 5. Conclusions

*E. coli* was the most frequently grown microorganism in our study, similar to previous years. In our study, microbial resistance to third- and fourth-generation cephalosporins developed, and the use of them in empirical therapy would lead to treatment failure. There was an increase in CoNS growth compared to previous years. Over the last decade, the culture results of this single-center study indicate that empirical antibiotic selection based on local microbial culture results and antibiotic susceptibility is crucial to allowing for more rational and successful use of antibiotics. The analysis of resistance risks in patient groups that demonstrated high resistance rates to third- and fourth-generation cephalosporins suggests that empirical treatment with alternative antibiotics showing higher susceptibility rates should be preferred, and that the antibiotic spectrum of action should be adjusted according to local culture data. The increase in these Gram-positive infections in SAI and the decrease in the susceptibility rates of quinolones and cephalosporins indicate that resistance to other antibiotic groups demonstrates the need to explore new antibiotics or our theoretical combination therapy for empirical treatment.

## Figures and Tables

**Table 1:** Demographic and clinical data in all cases.

All Cases	*n* = 291 (%)
**Age (years, mean)**	64 ± 12.87
Male	67 ± 13.3
Female	61.9 ± 12.1
**Sex**	
Male	173 (59.5)
Female	118 (40.5)
**Etiology of cirrhosis**	
Cryptogenic	108 (37.1)
HCV	71 (24.4)
HBV	57 (19.6)
Alcoholic liver disease	23 (7.9)
Primary biliary cirrhosis	4 (1.4)
Budd–Chiari	15 (5.1)
Other	13 (4.5)
**Child–Pugh score**	
Child A	1 (0.3)
Child B	78 (26.8)
Child C	212 (72.9)
**Culture results**	
CNNA	37 (12.7)
SBP	49 (16.8)
MNBA	23 (8)
Simple ascites	182 (62.5)
**Time to paracentesis in hospitalization**	
<24 h	237 (81.5)
24–48 h	38 (13.1)
48–72 h	10 (3.4)
>72 h	6 (2)
**Nosocomial infection**	-

CNNA: culture-negative neutrocytic ascites, SBP: spontaneous bacterial peritonitis, MNBA: monomicrobial non-neutrocytic bacterascites, HCV: hepatitis C, HBV: hepatitis B.

**Table 2:** SBP causative microorganisms.

Positive Culture Samples	SBP *n* = 49 (%)
**Types of microorganisms**	
*Escherichia coli*	23 (46.9)
Coagulase-negative staphylococci	7 (14.2)
Enterococci	5 (10.2)
*Streptococcus* spp.	4 (8.1)
Nonfermenting Gram-negative bacilli	1 (2)
*Acinetobacter baumani*	1 (2)
*Streptococcus pneumoniae*	3 (6.1)
*Klebsiella pneumoniae*	1 (2)
*Staphylococcus aureus*	2 (4)
*Acinetobacter lwoffii*	1 (2)
*Stenotrophomonas maltophilia*	1 (2)
*Listeria monocytogenes*	1 (2)
*Brucella*	1 (2)

**Table 3:** Distribution of resistant bacteria.

	SBP *n* = 11 (%)	MNBA *n* = 5 (%)
*Escherichia coli*	5 (45.5%)	-
Coagulase-negative staphylococci	4 (36.4%)	4 (80%)
*Klebsiella pneumoniae*	-	1 (20%)
*Acinetobacter baumannie*	1 (9.1%)	-
*Staphylococcus aureus*	1 (9.1%)	-

**Table 4:** Sensitivity of theoretical antibiotic combinations.

Antibiotic Combinations Sensitivity		Single Antibiotic Sensitivity		Single Antibiotic Sensitivity	
**Ampicillin/sulbactam** **+ Ciprofloxacin**	81%	**Ampicillin/** **sulbactam**	68%	**Ciprofloxacin**	66%
**Ampicillin/sulbactam** **+ Amikacin**	90%	**Ampicillin/** **sulbactam**	68%	**Amikacin**	90.3%
**Piperacillin/tazobactam + Ciprofloxacin**	94.4%	**Piperacillin/tazobactam**	81.8%	**Ciprofloxacin**	66%
**Piperacillin/tazobactam + Amikacin**	100%	**Piperacillin/tazobactam**	81.8%	**Amikacin**	90.3%
**Ceftriaxone + Ciprofloxacin**	86.4%	**Ceftriaxone**	70%	**Ciprofloxacin**	66%
**Ceftriaxone + Levofloxacin**	86.4%	**Ceftriaxone**	70%	**Levofloxacin**	71%
**Ceftriaxone + Amikacin**	96%	**Ceftriaxone**	70%	**Amikacin**	90.3%
**Cefepime + Amikacin**	96%	**Cefepime**	76.4%	**Amikacin**	90.3%
**Cefepime + Ciprofloxacin**	88.9%	**Cefepime**	76.4%	**Ciprofloxacin**	66%
**Cefepime + Levofloxacin**	88.5%	**Cefepime**	76.4%	**Levofloxacin**	71%

**Table 5:** Factors affecting mortality.

Factors	*p* Value	OR (95% CI)	Reference Category
Child–Pugh score	<0.001	–	–
Child A	0.950	0.0 (0–2.7)	Child C
Child B	<0.001	0.34 (0.24–0.50)	Child C
Child C	<0.001	–	Reference
Age	0.037	1.012 (1.00–1.02)	–
Sex	0.675	–	–
Polymicrobial growth	0.452	–	Monomicrobial growth
Multiple attacks	<0.001	1.62 (1.30–2.56)	Single episode
SAI	0.009	1.224 (0.91–1.63)	Sterile ascites
Etiology of cirrhosis	0.477	–	–
SBP	0.086	–	Non-SBP ascites

## Data Availability

All data generated or analyzed during this study are included in this published article. The authors confirm that the data are real, and you can receive them by consulting the corresponding author.
